# Pathological tau deposition in Motor Neurone Disease and frontotemporal lobar degeneration associated with TDP-43 proteinopathy

**DOI:** 10.1186/s40478-016-0301-z

**Published:** 2016-03-31

**Authors:** Roya Behrouzi, Xiawei Liu, Dongyue Wu, Andrew C. Robinson, Sayuri Tanaguchi-Watanabe, Sara Rollinson, Jing Shi, Jinzhou Tian, Hisham H. M. Hamdalla, John Ealing, Anna Richardson, Matthew Jones, Stuart Pickering-Brown, Yvonne S. Davidson, Michael J. Strong, Masato Hasegawa, Julie S. Snowden, David M. A. Mann

**Affiliations:** Clinical and Cognitive Sciences Research Group, Institute of Brain, Behaviour and Mental Health, Faculty of Medical and Human Sciences, University of Manchester, Salford Royal Hospital, Salford, M6 8HD UK; Beijing University of Chinese Medicine, Dongzhimen Hospital, 5 Hyancung St, Beijing, 100700 PR China; Department of Dementia and Higher Brain Function, Tokyo Metropolitan Institute of Medical Science, Setagaya-ku, Tokyo 156-8585 Japan; Clinical and Cognitive Sciences Research Group, Institute of Brain, Behaviour and Mental Health, Faculty of Medical and Human Sciences, University of Manchester, A V Hill Building, M13 9PT Manchester, UK; Manchester MND Care Centre, Salford Royal Hospital, Stott Lane, Salford, M6 8HD UK; Cerebral Function Unit, Greater Manchester Neurosciences Centre, Salford Royal Hospital, Stott Lane, Salford, M6 8HD UK; Molecular Brain Research Group, Robarts Research Institute, Western University, Canada, London, ON N6A 5B7 Canada

## Abstract

It has been suggested that patients with motor neurone disease (MND) and those with MND combined with behavioural variant frontotemporal dementia (bvFTD) (ie FTD + MND) or with FTD alone might exist on a continuum based on commonalities of neuropathology and/or genetic risk. Moreover, it has been reported that both a neuronal and a glial cell tauopathy can accompany the TDP-43 proteinopathy in patients with motor neurone disease (MND) with cognitive changes, and that the tauopathy may be fundamental to disease pathogenesis and clinical phenotype. In the present study, we sought to substantiate these latter findings, and test this concept of a pathological continuum, in a consecutive series of 41 patients with MND, 16 with FTD + MND and 23 with FTD without MND. Paraffin sections of frontal, entorhinal, temporal and occipital cortex and hippocampus were immunostained for tau pathology using anti-tau antibodies, AT8, pThr^175^ and pThr^217^, and for amyloid β protein (Aβ) using 4G8 antibody. Twenty four (59 %) patients with MND, 7 (44 %) patients with FTD + MND and 10 (43 %) patients with FTD showed ‘significant’ tau pathology (ie more than just an isolated neurofibrillary tangle or a few neuropil threads in one or more brain regions examined). In most instances, this bore the histological characteristics of an Alzheimer’s disease process involving entorhinal cortex, hippocampus, temporal cortex, frontal cortex and occipital cortex in decreasing frequency, accompanied by a deposition of Aβ up to Thal phase 3, though 2 patients with MND, and 1 with FTD did show tau pathology beyond Braak stage III. Four other patients with MND showed novel neuronal tau pathology, within the frontal cortex alone, specifically detected by pThr^175^ antibody, which was characterised by a fine granular or more clumped aggregation of tau without neurofibrillary tangles or neuropil threads. However, none of these 4 patients had clinically evident cognitive disorder, and this type of tau pathology was not seen in any of the FTD + MND or FTD patients. Finally, two patients, one with MND and one with FTD, showed a tau pathology consistent with Argyrophilic Grain Disease (AGD). Western blotting and use of 3- and 4-repeat tau antibodies confirmed the histological interpretation of Alzheimer’s disease type pathology in all instances except for those patients with accompanying AGD where a banding pattern on western blot, and immunohistochemistry, confirmed 4-repeat tauopathy. In all 3 patient groups, amyloid pathology was more likely to be present in patients dying after 65 years of age, and in the presence of *APOE* ε4 allele. We conclude that tau pathological changes are equally common amongst patients with MND, FTD + MND and FTD though, in most instances, these are limited in extent. In patients with MND, when cognitive impairment is present this is most likely due to an accompanying/evolving (coincidental) Alzheimer’s disease process or, as in a single case, Dementia with Lewy bodies, within the cerebral cortex rather than as a result of TDP-43 proteinopathy. Conversely, in FTD and FTD + MND dementia is more likely to be associated with TDP-43 proteinopathy than tau. Hence, present study shows no progression in severity of (tau) pathology from MND through FTD + MND to FTD, and does not support the concept of these conditions forming a continuum of clinical or pathological change.

**Electronic supplementary material:**

The online version of this article (doi:10.1186/s40478-016-0301-z) contains supplementary material, which is available to authorized users.

## Introduction

Motor Neurone Disease (MND), also known as Amyotrophic Lateral Sclerosis (ALS), is classically described as a neurodegenerative disorder of the locomotor system, characterised by degeneration and loss of upper and lower motor neurones, leading to a progressive weakness and wasting of limb, bulbar and trunk musculature, with death usually occurring within 2–3 years of symptom onset [3]. It affects 2–3 people in 100,000 worldwide, males slightly more than females. While about 90 % of cases appear to be sporadic in nature, with no known genetic cause, at least 6 genes are implicated in the pathogenesis of the remaining 10 % of familial cases [3]. These, in order of frequency, are expansions in *C9orf72*, and point mutations in *SOD-1*, *FUS*, *TARDBP*, *UBQLN1* and *VAPB* genes. In histological terms, all sporadic, and most familial cases (those associated with *C9orf72*, *TARDBP*, *UBQLN1* or *VAPB*), are characterised by the presence of neuronal cytoplasmic inclusions (NCI) within spinal and brainstem motor neurones composed of the TAR DNA binding protein of 43KDa, TDP-43, whereas cases associated with mutations in *SOD-1* and *FUS* display NCI within these same cell types containing these respective proteins [3].

However, MND is becoming increasingly recognised as a multisystem disorder in which behavioural changes and cognitive deficits can occur [12]. Cognitive change, particularly in executive functions, has been reported in up to half of patients [19, 28]. Of these, about 10–15 % patients fulfil criteria for behavioural variant frontotemporal dementia (bvFTD) [13, 28]. In keeping with the pattern of cognitive change, frontal lobe abnormalities have been demonstrated in MND both on structural [2, 17] and functional [1, 18] imaging. bvFTD may precede, follow or coincide with the onset of motor symptoms [24], reinforcing the inter-relationship between the two disorders.

The pathological substrate of dementia in MND, when combined clinically with bvFTD (henceforth termed FTD + MND), has been consistently linked to TDP-43 rather than tau pathology [4, 26]. However, the basis for changes underlying cognitive deficits in MND, which do not match up to fully fledged bvFTD, remains unclear. It is of interest, therefore, that in a recent study, Yang and Strong [36] found evidence of both TDP-43 and tau pathology in MND patients with and without cognitive impairment. These authors employed novel tau polyclonal antibodies to investigate tau pathology in 10 patients with clinically and pathologically confirmed Amyotrophic Lateral Sclerosis (ALS) (aka MND). Five showed cognitive impairment (ALSci), as defined by Strong [31], whereas five showed no cognitive impairment. In patients with ALS alone, an antibody directed against tau phosphorylated at Thr175 (pThr^175^) detected limited neuronal tau aggregates predominantly within entorhinal cortex and amygdala, whereas an antibody directed against tau phosphorylated at Thr217 (pThr^217^) detected astrocytic tau deposition in frontal cortex as well as in entorhinal cortex and amygdala. In patients with ALSci, a more extensive spread of neuronal pThr^175^ was seen, involving the frontal lobe, whereas for pThr^217^ a more extensive astrocytic involvement than in MND alone was observed. These findings prompted Yang and Strong [34] to suggest that in patients with MND with cognitive changes, a coincidental tau and TDP-43 pathology is present, and that widespread (astrocytic) tau pathology may be fundamental to pathogenesis. Furthermore, Bieniek et al [6] noted excessive tau pathology in a higher proportion of patients with FTLD-TDP associated with an expansion in *C9orf72*, and in others with FTLD-TDP with no known mutation, when compared to cases of FTLD-TDP with *GRN* mutations and suggested that some forms of TDP-43 proteinopathy might favour or promote the development of tauopathy. Hence, the overlap between TDP-43 and tau pathologies in ALS [36] and FTLD [6], and the more marked tau pathology in patients with MND with, rather than without, cognitive impairment [36], could be interpreted as supporting the spectrum/continuum notion of the relationship between ALS and bvFTD.

If this were so, it might be postulated that the clinical combination of FTD + MND could be driven in either direction from FTD or from MND through a common pathogenetic pathway. By this argument, it might be anticipated that tauopathy in MND would be exacerbated in FTD + MND, and even more so in FTD. In order to test this hypothesis we have used the same tau polyclonal antibodies used by Yang and Strong [36] to evaluate tau pathology in an independent cohort of patients with MND, as well as in patients with FTD + MND and those with FTD without MND.

## Methods

### Patients

The study group consisted of 80 patients, 41 with a clinical diagnosis of MND (27 males, 14 females; patients #1–41), 16 clinically diagnosed with FTD + MND (10 males, 6 females; patients #42–57) and 23 patients with FTD but without MND (15 males, 8 females; patients #58–80) (Table 1). Fifteen of the FTD group of patients had a predominantly bvFTD phenotype whereas the other 8 patients had a predominant language phenotype (Table 1); for purposes of comparison all were subsumed under the rubric of FTD without MND. Notably, all 23 patients within this FTD group shared a common TDP-43 histological phenotype (see below). No patients were available in which the clinical syndromes of Progressive Non-Fluent Aphasia (PNFA) or Semantic Dementia were combined with MND. The brains of these patients were consecutively acquired by the Manchester Brain Bank over the years 1986 to 2015. All patients were from the North West of England and North Wales and tissues were obtained through appropriate consenting procedures for the collection and use of the human brain tissues. The 16 patients with FTD + MND and the 23 patients with FTD without MND fulfilled relevant clinical diagnostic criteria [14, 25, 27]. They had all been investigated longitudinally within a specialist dementia clinic using the Manchester Neuropsychological Profile (Man-NP) [30, 34] to determine and characterise the nature of their dementia. Some of the MND patients had also undergone this formal neuropsychological assessment, though in most others where this had not been performed the presence of cognitive impairment was deduced (in patients #35 and 36) from inspection of clinical notes and medical correspondence by specialist neuropsychologists. All 41 patients with MND fulfilled El Escorial criteria [9].

**Table 1:** Selected clinical, neuropathological and genetic details on patients studied

Case ID#	MRC#	Clinical diagnosis	Cognitive status	Pathological diagnosis	Gender	Onset age	Age at death	Duration	APOE genotype	Family history	Mutation	Thal stage	Braak stage
1	na	MND	unknown	MND	F	56	57	1	na	N	na	0	0
2	na	MND	unknown	MND	F	50	52	2	na	N	na	0	0
3	na	MND	unknown	MND	M	77	77	0.5	na	N	na	0	0
4	na	MND	unknown	MND	M	na	na	na	na	na	na	0	0
5	na	MND	unknown	MND	M	na	na	na	na	na	na	0	0
6	na	MND	unknown	MND	M	na	na	na	na	na	na	0	0
7	BBN_3043	MND	unknown	MND	M	42	45	2	33	N	none	0	0
8	BBN_3068	MND	normal	MND	M	60	63	3	na	N	na	0	0
9	BBN_3158	MND	unknown	MND	M	na	na	na	33	N	none	0	0
10	BBN_3340	MND	normal	MND	M	43	45	2	33	Y	C9orf72	0	0
11	BBN_3436	MND	normal	MND	M	49	51	2	33	N	none	0	0
12	BBN_6065	MND	normal	MND	M	53	55	2	33	N	none	0	0
13	BBN_18408	MND	normal	MND	M	40	46	6	34	N	none	0	0
14	BBN_24300	MND	normal	MND	M	55	58	3	33	N	C9orf72	0	0
15	na	MND	unknown	MND	F	69	71	2	na	N	na	3	0
16	na	MND	unknown	MND	F	na	na	na	na	na	na	1	0
17	BBN_3307	MND	normal	MND	M	66	72	6	33	N	none	1	0
18	na	MND	unknown	MND	M	42	44	?	na	N	na	0	0-I
19	BBN_3106	MND	unknown	MND	F	72	76	4	33	N	none	0	0-I
20	BBN_3268	MND	normal	MND	M	na	na	na	33	N	none	0	0-I
21	BBN_3317	MND	normal	MND	M	na	na	na	33	N	none	0	0-I
22	BBN_3429	MND	normal	MND	M	50	52	2	34	N	none	0	0-I
23	BBN_3456	MND	normal	MND	F	55	57	2	23	N	none	0	I
24	BBN_10257	MND	unknown	MND	M	74	77	3	33	N	none	0	I
25	BBN_3315	MND	normal	MND	M	65	67	2	33	N	none	0	I
26	BBN_24685	MND	normal	MND	F	53	58	5	34	N	none	0	I
27	BBN_3319	MND	normal	MND	F	na	na	na	33	N	none	3	0-I
28	BBN_3330	MND	normal	MND	F	na	na	na	33	N	none	2	0-I
29	na	MND	unknown	MND	M	na	na	na	na	na	na	3	I
30	BBN_3212	MND	normal	MND	M	60	65	5	34	Y	C9orf72	3	I-II
31	BBN_3341	MND	normal	MND	F	83	85	1.5	34	N	none	5	I-II
32	BBN_3342	MND	normal	MND	F	65	67	2	33	N	none	3	I-II
33	na	MND	unknown	MND	M	na	na	na	na	na	na	3	II
34	BBN_25502	MND	normal	MND	F	73	76	3	33	N	none	3	II
35	BBN_13803	MND	dementia	MND	F	65	69	4	23	N	none	3	IV
36	BBN_3297	MND	cognitive impaired	MND	F	na	na	na	33	N	none	3	IV-V
37	BBN_3004	MND	normal	MND	M	43	47	4	na	N	na	0	na
38	BBN_3344	MND	normal	MND	M	55	57	2	33	Y	C9orf72	0	na
39	BBN_3418	MND	normal	MND	F	37	39	2	33	N	none	0	na
40	BBN_6078	MND	normal	MND	M	46	52	4	34	N	none	2	na
41	BBN_3457	MND	normal	MND	M	60	63	3	33	N	none	0	na
42	BBN_5658	FTD+MND	dementia	FTLD-TDP B	M	45	47	2	na	N	na	0	0
43	BBN_5661	FTD+MND	dementia	FTLD-TDP B	M	43	45	2	34	N	none	0	0
44	BBN_5663	FTD+MND	dementia	FTLD-TDP B	M	57	59	2	33	Y	C9orf72	0	0
45	BBN_5669	FTD+MND	dementia	FTLD-TDP B	M	65	67	2	33	N	none	0	0
46	BBN_5732	FTD+MND	dementia	FTLD-TDP B	F	50	52	3	34	Y	none	0	0
47	BBN_3334	FTD+MND	dementia	FTLD-TDP B	M	na	na	na	33	N	none	0	0
48	BBN_3459	FTD+MND	dementia	FTLD-TDP B	F	61	63	2	33	N	none	0	0
49	BBN_10258	FTD+MND	dementia	FTLD-TDP B	M	61	64	3	33	N	none	0	0
50	BBN_14791	FTD+MND	dementia	FTLD-TDP B	M	72	75	3	34	N	none	0	0
51	BBN_24314	FTD+MND	dementia	FTLD-TDP B	F	59	63	4	33	N	none	0	0-I
52	BBN_5764	FTD+MND	dementia	FTLD-TDP B	M	61	65	4	33	N	none	0	0-I
53	BBN_5772	FTD+MND	dementia	FTLD-TDP B	F	70	73	3	33	Y	C9orf72	0	0-I
54	BBN_24359	FTD+MND	dementia	FTLD-TDP B	M	51	58	7	33	Y	C9orf72	0	0-I
55	BBN_24376	FTD+MND	cognitive impaired	FTLD-TDP B	F	78	79	1	34	N	none	0	0-I
56	BBN_5771	FTD+MND	dementia	FTLD-TDP B	F	63	65	2	33	Y	C9orf72	0	I
57	BBN_5721	FTD+MND	dementia	FTLD-TDP B	M	58	69	11	33	N	none	1	I
58	BBN_5681	FTD	dementia	FTLD-TDP A	M	49	58	9	33	Y	C9orf72	0	0
59	BBN_5705	FTD	dementia	FTLD-TDP A	F	53	67	14	33	Y	V452WfsX38	0	0
60	BBN_10260	PNFA	dementia	FTLD-TDP A	M	62	72	10	33	Y	V452WfsX38	0	0
61	BBN_5660	FTD	dementia	FTLD-TDP A	F	53	71	18	33	Y	V452WfsX38	0	0
62	BBN_5773	FTD	dementia	FTLD-TDP A	M	66	73	7	33	Y	Q130SfsX124	0	0
63	BBN_5715	PNFA	dementia	FTLD-TDP A	F	63	71	8	33	Y	Q130SfsX124	0	0
64	BBN_5718	FTD	dementia	FTLD-TDP A	M	59	66	7	33	Y	R493X	0	0
65	BBN_5675	FTD	dementia	FTLD-TDP A	F	51	61	10	na	Y	R493X	0	0
66	BBN_5686	FTD	dementia	FTLD-TDP A	F	60	66	6	33	N	Q468X	0	0
67	BBN_5734	FTD	dementia	FTLD-TDP A	M	69	75	6	33	N	none	0	0
68	BBN_5757	PNFA	dementia	FTLD-TDP A	F	66	77	11	33	N	none	0	0
69	BBN_5666	PNFA	dementia	FTLD-TDP A	M	55	71	16	34	Y	V452WfsX38	2	0
70	BBN_5677	PNFA	dementia	FTLD-TDP A	M	62	70	8	34	Y	V452WfsX38	2	0
71	BBN_5719	FTD	dementia	FTLD-TDP A	F	59	64	5	33	Y	C9orf72	0	0-I
72	BBN_5752	FTD	dementia	FTLD-TDP A	M	64	72	8	33	Y	C9orf72	0	0-I
73	BBN_5739	FTD	dementia	FTLD-TDP A	M	62	67	5	33	Y	C9orf72	0	0-I
74	BBN_14793	FTD	dementia	FTLD-TDP A	M	54	65	11	33	Y	C9orf72	0	0-I
75	BBN_5727	PNFA	dementia	FTLD-TDP A	M	66	73	7	33	Y	C31LfsX34	0	0-I
76	BBN_5742	PNFA	dementia	FTLD-TDP A	M	66	71	5	34	Y	V452WfsX38	0	I
77	BBN_5774	FTD	dementia	FTLD-TDP A	M	63	65	2	34	Y	C9orf72	2	0-I
78	BBN_5706	FTD	dementia	FTLD-TDP A	M	60	68	8	34	Y	C9orf72	2	0-I
79	BBN_5685	PNFA	dementia	FTLD-TDP A	M	68	78	10	34	N	none	3	IV-V
80	BBN_5753	FTD	dementia	FTLD-TDP A	F	66	72	6	33	N	none	3	I-II

Comparison of the three patient groups showed no significant differences in gender distribution (χ^2^ = 0.095, *p* = 0.953) or mean age at onset of disease (F_2,65_ = 0.89, *p* = 0.416). However, mean age at death and duration of illness did differ (F_2,65_ = 6.0, *p* = 0.004, F_2,65_ = 33.8, *p* < 0.001, respectively). Patients with FTD alone died at a later age than those with MND (*p* = 0.003) and both patients with MND, and those with FTD + MND, had a shorter disease duration than those with FTD alone (*p* < 0.001), though those with MND and FTD + MND did not differ in this respect (Table 2).

**Table 2:** Mean (±SD) values for age at onset of symptoms, age at death and duration of illness for patients with Motor Neurone Disease (MND), behavioural variant Frontotemporal Dementia and Motor Neurone Disease (FTD + MND) and FTD. Also shown are mean (±SD) values for age at onset of symptoms, age at death and duration of illness for those cases of MND, FTD + MND and FTD, collectively, with mutations in *GRN*, expansion in *C9orf72*, or no known mutation, along with mean (±SD) values for age at onset of symptoms, age at death and duration of illness for those cases of MND, FTD + MND and FTD, collectively, showing with and without amyloid pathology, and those with and without (any type of) tau pathology

Group	Age at onset (y)	Age at death (y)	Duration of illness (y)
MND (*n* = 41)	57.2 ± 12.2	60.1 ± 12.0*	2.9 ± 1.4**
FTD + MND (*n* = 16)	59.8 ± 9.4	63.1 ± 9.4	3.4 ± 2.4**
FTD (*n* = 23)	60.7 ± 5.7	69.3 ± 4.9	8.8 ± 3.7
*GRN* mutation (*n* = 12)	59.7 ± 5.5	69.3 ± 3.6	9.7 ± 4.2
*C9orf72* expansion (*n* = 15)	57.7 ± 7.1	62.9 ± 7.1	5.2 ± 3.0!!!
No genetic mutation (*n* = 53)	59.2 ± 11.4	62.7 ± 11.8	3.5 ± 2.4!!!
All cases with amyloid (*n* = 21)	64.2 ± 8.1$$	70.0 ± 7.2$$$	5.8 ± 4.2
All cases without amyloid (*n* = 59)	57.5 ± 9.8	62.2 ± 10.4	4.7 ± 3.5
All cases with tau (*n* = 41)	60.7 ± 10.4	65.1 ± 10.5	4.4 ± 2.7
All cases without tau (*n* = 39)	57.4 ± 9.0	60.7 ± 10.4	5.4 ± 4.4

*, ** indicate significantly different from FTD group, *p* = 0.003 and *p* < 0.001, respectively

!!! indicates significantly different from *GRN* mutation group, *p* < 0.001

$$, $$$ indicate significantly older than cases without amyloid, *p* = 0.003, <0.001, respectively

Four patients with MND (patients #10, 14, 30 and 38), 4 with FTD + MND (patients #44, 53, 54 and 56) and 7 with FTD (patients #58, 71–74, 77 and 80) bore an expansion in *C9orf72*, as evidenced by Southern blot and/or repeat primed PCR [11, 20] (Table 1). Twelve of the other patients with FTD (patients #59–66, 69, 70, 75 and 76) bore mutation in progranulin gene (*GRN*). No mutation was known to be present in the remaining 4 patients (patients # 67, 68, 79 and 80) (Table 1). There were no significant differences between age at onset (F_2,65_ = 0.158, *p* = 0.854) or age at death (F_2,65_ = 2.10, *p* = 0.130) between carriers of *GRN* mutation, *C9orf72* expansion or those with no known mutation, though duration of illness did vary significantly between the three groups (F_2,65_ = 21.2, *p* < 0.001) with bearers of *GRN* mutation having a significantly longer disease course than either those with *C9orf72* expansion or those without known mutation (*p* < 0.001 in both instances), which did not differ from each other (*p* = 0.140) (Table 2).

Previous pathological diagnostic investigations had shown all MND and FTD + MND patients to display atrophy and loss of motor neurones from trigeminal and hypoglossal cranial nerve nuclei, and anterior horn cells (where spinal cord was available), with the presence of skein-like, or rounded, more solid, TDP-43 immunoreactive neuronal cytoplasmic inclusions (NCI) within surviving cells, or with fine, particulate accumulations of TDP-43, in which the nucleus has been ‘cleared’ of its normal immunoreactivity Additional file 1: Figure S1. Patient #35 with MND also had isocortical DLB [22], along with Alzheimer-type pathology, though typical TDP-43 pathology was still seen in anterior horn cells of the spinal cord (see Additional file 1: Figure S1). Thirty four MND patients showed no extramotor TDP-43 pathology at all, whereas 7 MND patients showed occasional or moderate numbers of NCI within dentate gyrus granule cells, four of whom also displayed moderate numbers of, or many, TDP-43 immunopositive granules within the cytoplasm of small pyramidal cells of layer II of the frontal and temporal cortex, though well-formed NCI were only rarely present. On the other hand, all 16 patients with FTD + MND showed widespread TDP-43 immunoreactive NCI within hippocampal dentate gyrus granule cells and numerous cells in layer II of the frontal and temporal cortex contained TDP-43 immunopositive granules with well-formed NCI in others, in the relative absence of TDP-43 immunoreactive neurites, consistent with neuropathological classification of FTLD-TDP type B [20]. Additionally, there was loss of motor neurones from trigeminal and hypoglossal cranial nerve nuclei, and anterior horn cells (where spinal cord was available) with TDP-43 immunoreactive NCI within surviving cells. Conversely, all 23 patients with FTD alone showed numerous TDP-43 immunoreactive NCI and neurites in layer II of the frontal and temporal cortex with variable numbers of TDP-43 immunoreactive NCI in granule cells of the dentate gyrus of the hippocampus, consistent with pathological classification of FTLD-TDP type A [20]. Additionally, those patients bearing *GRN* mutations showed variable presence of TDP-43 immunoreactive neuronal intranuclear inclusions (NII) in neurones of layer II of frontal and temporal cortex, but these were not seen in those patients bearing expansion in *C9orf72*, or in the 4 cases without known mutation. Dipeptide repeat proteins consisting of poly-GA, poly-GP and poly-GR proteins were present in CA4 neurones of hippocampus and granule cells of the dentate gyrus and cerebellum in all 16 *C9orf72* expansion bearers, irrespective of clinical phenotype [11].

### Immunohistochemistry

Paraffin sections were cut at 6 μm from formalin fixed blocks of frontal lobe (BA8/9), temporal lobe (BA21/22) including anterior and posterior hippocampus and entorhinal cortex, occipital lobe (BA17/18), corpus striatum and cerebellum from all individuals. We did not include sections from ‘neighboring’ areas such as insular and cingulate cortex into the study as previous diagnostic neuropathological analyses had not revealed these to be different (in terms of tau pathology) from chosen areas of temporal and frontal cortex, respectively. Following titration to determine optimal immunostaining, antibodies were identically employed in a standard IHC protocol, as described previously [11, 21]. Frontal, temporal (to include hippocampus and entorhinal cortex) and occipital lobe sections were immunostained for tau proteins. The following tau antibodies were employed: AT8 (1:750), pThr^175^ and pThr^217^ (both of which were used at 1:1000 dilution). These latter antibodies are polyclonal phospho-tau antibodies generated against sequences Ac-SLP[pT]PPTREPC-amide and Ac-RIPAK[pT]PPAPKC-amide, respectively. Full details regarding the production and specificity of these antibodies have been presented elsewhere [36]. Negative controls omitting pThr^175^ and pThr^217^ antibody, and normal brain sections known to be free from tau pathology using AT8 antibody were employed to substantiate the specificity of pThr^175^ and pThr^217^ antibodies. Selected sections of frontal and temporal cortex (see later) were immunostained for 3-repeat (3-R) and 4-repeat (4-R) tau proteins using RD3 and RD4 antibodies (Millepore), at a dilution of 1:1500 and 1:200, respectively. For each tau antibody, antigen unmasking was performed by pressure cooking in citrate buffer (pH 6.0, 10 mM) for 30 min, reaching 120° Celsius and >15 kPa pressure. Additional sections of frontal, temporal (to include hippocampus and entorhinal cortex) and occipital cortex, along with those of corpus striatum and cerebellum, were immunostained for amyloid plaques using 4G8 antibody (1:3000). Antigen retrieval was in this case performed by immersion in 95 % formic acid for 5 min prior to incubation in primary antibody. Sections of frontal and temporal cortex were also immunostained for TDP-43 and phosphorylated α-synuclein as above.

AT8, pThr^175^ and pThr^217^ immunostained sections were scored microscopically at an objective magnification of x25 (overall magnification of x250) for the presence and severity of tau pathological changes, as visualised by each of the tau antibodies, employing the following rating scale:

- 0 = No tau pathology present.
- 0.5 = rare (ie 1–5 tau immunoreactive neurofibrillary tangles/neurites per section.
- 1 = 1–5 tau immunoreactive neurofibrillary tangles/neurites per x250 microscope field.
- 2 = 5–10 tau immunoreactive neurofibrillary tangles/neurites per x250 microscope field.
- 3 = more than 10 tau immunoreactive neurofibrillary tangles/neurites per x250 microscope field.

Cases were also assessed for the extent and distribution of neurofibrillary (AT8) and amyloid plaque (4G8) pathology, employing Braak and Braak [7] and Thal [33] staging procedures, respectively. Cases where no tau pathology whatsoever was present were staged 0, those where only rare neurofibrillary tangles were present in entorhinal cortex alone were staged 0-I. Stage I cases showed abundant tangles in entorhinal cortex alone [see 7]. Neuritic plaques were rated according to CERAD criteria.

### Western blotting

200–500 mg samples of frozen frontal and temporal cortex were dissected from selected tau-immunopositive cases (see later) and subjected to western blot analysis of insoluble tau, as we have described elsewhere [32]. Briefly, sarkosyl-insoluble pellets were prepared by homogenization of tissue samples in 20vol (v/w) of extraction buffer containing 10 mM Tris–HCl (pH 7.5), 0.8 M NaCl, 10 % sucrose, 1 mM EGTA, 2 % sarkosyl and incubated for 30 min at 37 °C. After centrifugation at 20,000 g for 10 min at 25 °C, the supernatants were taken, transferred to 1.5 mL tubes and ultracentrifuged at 100,000 g for 20 min at 25 °C. The pellets were washed by ultracentrifugation with 0.5 mL of sterile saline, solubilized in SDS-sample buffer and subjected to 4–20 % gradient polyacrylamide gel (Wako) SDSPAGE. Proteins were transferred to PVDF membrane, incubated overnight with the anti-tau monoclonal antibody T46 (Thermo Scientific), biotinylated 2nd antibody, avidin–biotin complex (Vector) and developed with diaminobenzidine and nickel chloride.

### Statistical analysis

Comparisons of semiquantitative scores for severity of AT8, pThr^175^ and pThr^217^ immunostaining in frontal and temporal cortex, entorhinal cortex and CA1 region of hippocampus, were performed using Kruskal-Wallis test with post-hoc Mann-Whitney test where Kruskal-Wallis yielded a significant difference between antibody staining scores. Comparisons of *APOE* ε4 allele frequency between MND, FTD + MND and FTD groups, and cases of MND, FTD + MND and FTD, collectively, with and without amyloid deposition, were made using Chi squared test. Comparisons of mean age at onset, age at death and duration of illness between patients with MND, FTD + MND and FTD, with and without amyloid deposition, were made using unpaired t-test. Significance levels were set at *p* < 0.05 throughout.

All research reported in the paper was performed with ethical approval under the Manchester Brain Bank Generic Tissue Bank Ethics approved by Newcastle and North Tyneside Ethics Committee.

## Results

### Tau immunostaining

Overall, when using the full panel of tau antibodies (ie AT8, pThr^175^ and pThr^217^ antibodies), 17 (41 %) patients with MND (patients #1–17), 9 (56 %) patients with FTD + MND (patients #42–50) and 13 (56 %) patients with FTD alone (patients #58–70) showed either no tau pathology at all, or only isolated neuronal tau pathology, in any region of brain examined. These 39 patients were classed as Braak stage 0.

Eleven patients (27 %) with MND (patients #18–25 and #27–29), 7 patients (44 %) with FTD + MND (patients #51–57) and 9 with FTD (39 %) showed sparse tau neuronal pathology (ie a single or a few neurofibrillary tangles and/or a few neuropil threads per section, usually only in a single brain region, and then most often in the entorhinal cortex) with any, or all, of the 3 tau antibodies employed. These 27 patients were classed as Braak stage 0-I/I.

The remaining 13 patients (31 %) with MND (patients #26, 30–41) (but none with FTD + MND and only 1 with FTD (patient #79 with PNFA) displayed a ‘significant degree of tau pathology’, as defined by the presence of a few to many neurofibrillary tangles and/or neuropil threads in several brain regions, usually with all 3 tau antibodies and again usually to a similar extent and with a similar distribution. Eight of these patients (patients #26, 30–36 and 79) were classed as having Braak stages II and greater. Seven patients (patients #30–36) showed moderate to severe involvement of entorhinal cortex with mild to severe involvement of CA1 region of hippocampus, but this was without neocortical involvement in patients #30–34, consistent with Braak stages I-II/II. The other 3 patients (patients #35, 36 and 79) (see Additional file 2 for full clinical and neuropathological details of patient #35) also showed moderate or severe involvement of inferior temporal gyri and superior frontal cortex (Braak stage IV), and 2 of these (patients #36 and 79) had some involvement of the visual association cortex, but not primary visual cortex, consistent with Braak stages IV-V. The pattern of tau pathology in the remaining 5 MND patients (patients #37–41) was such that it was not possible to Braak stage these cases (see later). Patients with MND were no more, or no less, likely to display some/any degree of tau pathology than those with FTD + MND, or those with FTD (χ2 = 0.037, *p* = 0.982).

**Fig. 1:**
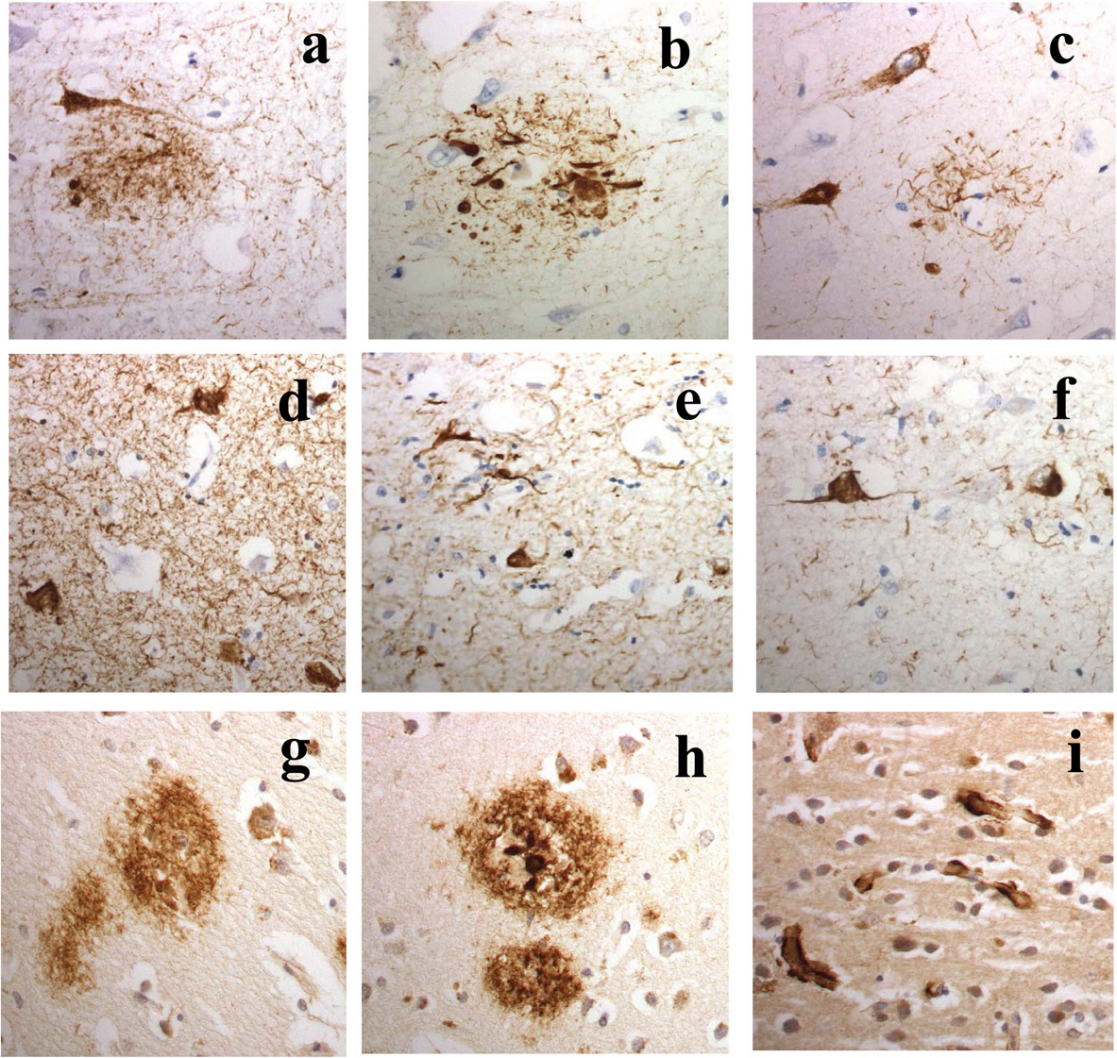
Tau and amyloid pathology in MND Group 1 patients. Alzheimer-type tau pathology is present in CA1 region of hippocampus (**a**-**c**) and entorhinal cortex (**d**-**f**) in patient #35 with MND and Dementia with Lewy bodies as shown by immunostaining for tau with AT8 (**a** and **d**), pThr175 (**b** and **e**) and pThr217 (**c** and **f**) antibodies. Both diffuse (**g**) and cored (**h**) amyloid plaques are present with 4G8 antibody. Cerebral amyloid angiopathy affecting capillaries (**i**) is present in patient #33. Immunoperoxidase x400

Three major patterns of tau pathology were noted, and patients were grouped accordingly. Group 1 tau staining pattern was most common, irrespective of the actual amount of staining present, being seen in 19 (of the 24 tau positive) patients with MND (patients #18–36), in all 7 patients with FTD + MND (patients #51–57) and in 9 of the patients with FTD (patients #71–79). The staining pattern resembled that of an Alzheimer’s disease-type process (Fig. 1a-f), with a few to many neurofibrillary tangles and neuropil threads being present within entorhinal cortex (in all 19 tau-positive patients with MND, all 7 tau-positive patients with FTD + MND and all 10 tau-positive patients with FTD), CA1 region of hippocampus (16/17 patients with MND, all 7 patients with FTD + MND and 8/9 patients with FTD), inferior and middle temporal gyri (15/17 patients with MND, 4/7 patients with FTD + MND and 5/9 patients with FTD), superior frontal cortex (9/17 patients with MND, 1/5 patients with FTD + MND and 5/9 patients with FTD) and visual association cortex (1/17 patients with MND and 1/9 patients with FTD) (see Fig. 1). Interestingly, 4 patients with MND patients (patients #19, 20, 25 and 26) also showed extensive CA2 tau pathology.

**Fig. 2:**
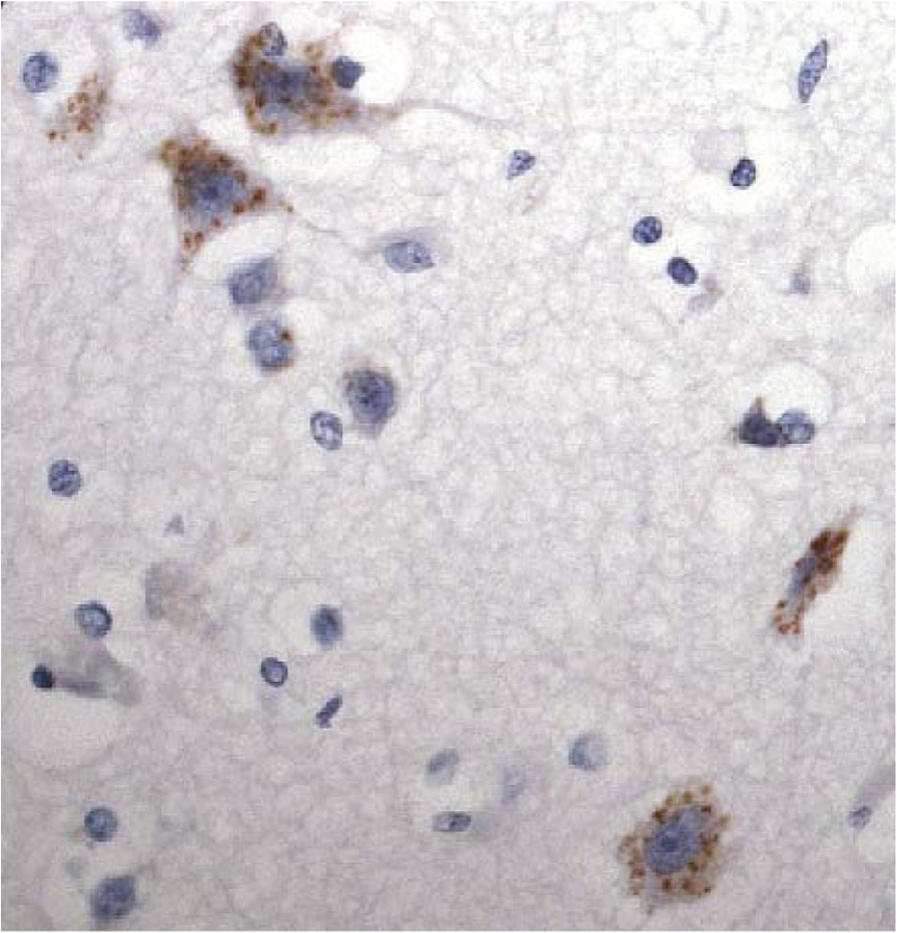
Tau pathology in MND group 2 patients. Neuronal tau pathology is present in frontal cortex in patient #38, and detected by pThr175 antibody alone. Immunoperoxidase x400

Group 2 tau staining pattern was seen in 4 patients (patients #37–40) (Fig. 2). Here, there was occasional to frequent tau immunostaining of neurones of superior frontal cortex, and to a lesser extent inferior temporal cortex, but no involvement of entorhinal cortex, occipital cortex or CA1 region of hippocampus, with pThr^175^ antibody: no such immunostaining was seen with either AT8 or pThr^217^ antibodies. In contrast to the above group of patients, the tau immunostaining appeared either finely, or coarsely, granular with no neurofibrillary tangle-like structures, or neuropil threads, being seen. Although one of the patients showing the group 2 form of tau pathology (patient #38) bore an expansion in *C9orf72*, the tau pathology did not appear to be specifically associated with this genetic change as none of the other 3 patients with this tau particular pathology bore an expansion in *C9orf72*, nor did any of the other 7 expansion carriers display group 2 type changes in tau.

**Fig. 3:**
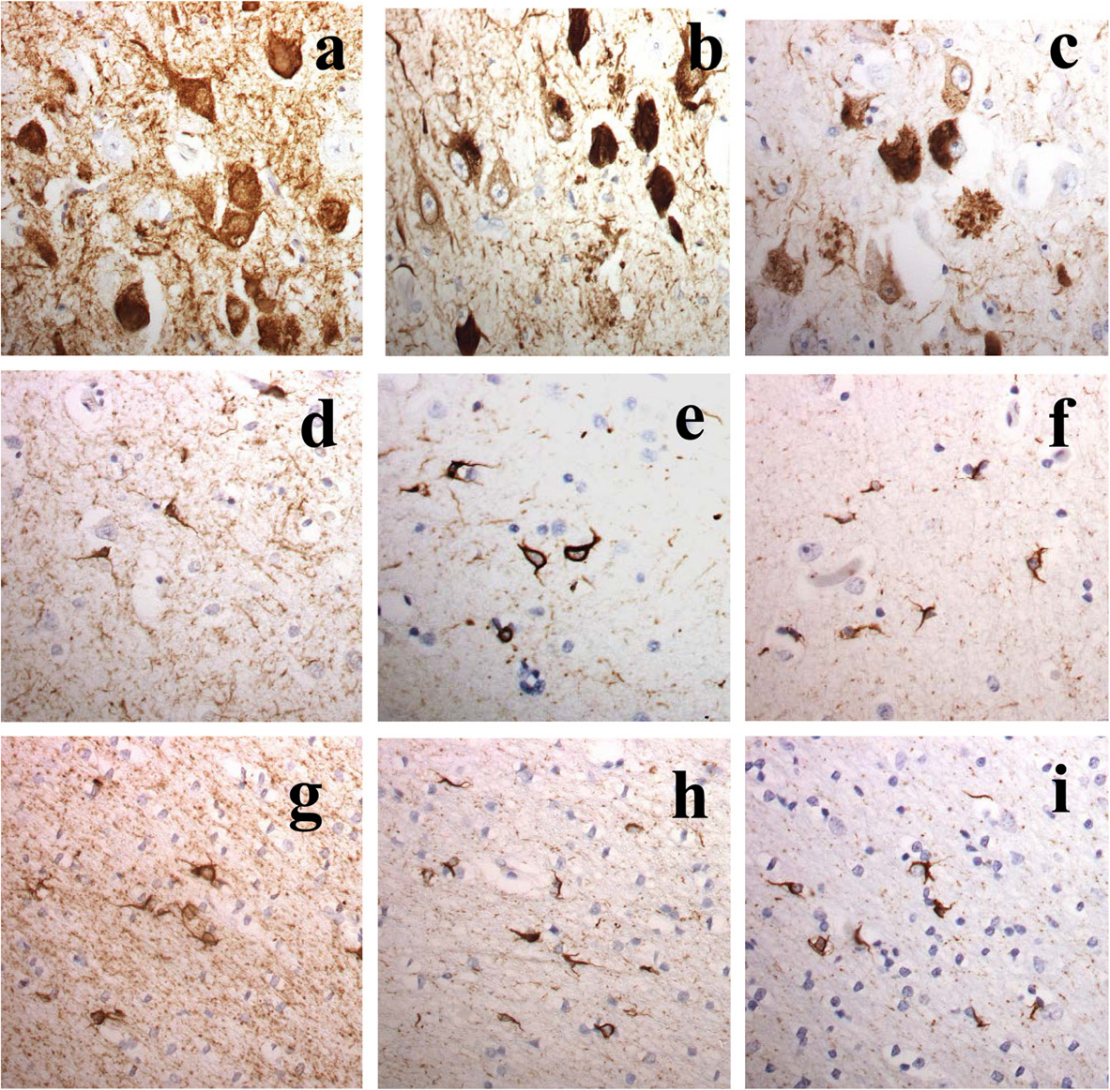
Tau pathology in Group 3 patients. Neuronal and neuritic plaque (**a**-**c**) tau pathology in CA1 region of hippocampus (**a**-**c**) and glial cell (**d**-**i**) tau pathology in entorhinal cortex grey matter (**d**-**f**) and white matter (**g**-**i**) as shown by immunostaining with AT8 (**a**, **d** and **g**), pThr175 (**b**, **e** and **h**) and pThr217 (**c**, **f** and **i**) antibodies. Immunoperoxidase x400

In patients #41 (see Additional file 2 for full clinical and neuropathological details) and 80 (group 3), a third pattern of tau pathology was seen (Fig. 3). In this there was mild neurofibrillary tangle formation in granule cells of the dentate gyrus of the hippocampus, and in areas CA3 and CA4. However, there was total involvement of CA2 region with all cells being affected by neurofibrillary tangles or containing amorphous tau but without apparent cell loss. There was severe loss of cells from CA1 and subiculum, with severe hippocampal sclerosis, with the remainder containing neurofibrillary tangles. Likewise the entorhinal cortex was severely affected (especially layer II stellate cells) and this extended into layers III and V of the adjoining inferior temporal gyrus, thinning out to minimal involvement in superior temporal gyrus, and superior frontal gyrus. In addition to the neuronal pathology, there was dense oligodendroglial cell involvement in the form of tangles resembling coiled bodies. These were most numerous in white matter in entorhinal cortex and inferior temporal gyrus, becoming infrequent in superior temporal and superior frontal gyri. This pattern of tau pathology was consistent with Argyrophilic Grain Disease (AGD).

In some patients, occasional glial cells, resembling astrocytes, also showed some granular, or fibrillary, tau immunoreactivity with all 3 tau antibodies, though for the most part this did not adopt a consistent pattern, nor was it present in anything but isolated cells. Notably, we did not observe any specific immunostaining of glial cells of the kind described by Yang and Strong [34] using pThr^217^ antibody in any patient.

### Comparisons between immunostaining with AT8, pThr^175^ and pThr^217^ antibodies

Semiquantitative scores for tau pathology, as detected by AT8, pThr^175^ and pThr^217^ antibodies, were compared in each of entorhinal cortex, CA1 region of hippocampus, temporal and frontal neocortex by Kruskal-Wallis test. No significant difference between the degree of tau antibody staining was detected for CA1 region, entorhinal cortex, temporal cortex, or frontal cortex either when all 80 patients were grouped together, or when split according to clinical grouping (Table 3). Patients were also grouped according to their pattern of tau pathology (as described above) including, as group 4, those patients with no or isolated tau (ie patients #1–17, #42–50 and #58–70). Again, no significant difference between the degree of tau staining with each of the three antibodies was detected for CA1 region, entorhinal cortex, temporal cortex, or frontal cortex for tau groups 1, 3 and 4. However, for tau group 2 there was a significant difference between the degree of tau antibody staining in frontal cortex (χ2 = 10.51, *p* = 0.005), but not in the other 3 regions (Table 3). Post hoc analysis showed that the level of tau staining was significantly higher with pThr^175^ than with pThr^217^ (*p* = 0.029) or AT8 (*p* = 0.029) antibodies, but the latter 2 did not differ significantly (*p* = 0.999), thereby bearing out microscopic observations.

**Table 3:** Significance values for Kruskal-Wallis comparisons of degree of AT8, pThr^175^ and pThr^217^ tau immunostaining in CA1 region of hippocampus, entorhinal cortex, temporal cortex and frontal cortex for all 80 patients, collectively or when stratified according to clinical phenotype, or tau pathological profile

Group	CA1	Entorhinal cortex	Temporal cortex	Frontal cortex
All cases (*n* = 80)	0.726	0.544	0.834	0.877
MND (*n* = 41)	0.758	0.518	0.963	0.371
FTD + MND (*n* = 16)	0.675	0.577	0.231	0.993
FTD (*n* = 23)	0.935	0.753	0.717	0.544
Tau group 1 (*n* = 35)	0.621	0.607	0.759	0.539
Tau group 2 (*n* = 4)	1.000	0.368	0.113	**0.005**
Tau group 3 (*n* = 2)	0.535	0.852	0.275	0.882

bolded figures indicate significant difference in staining between the three anti-tau antibodies

### Tau isoform analysis

**Fig. 4:**
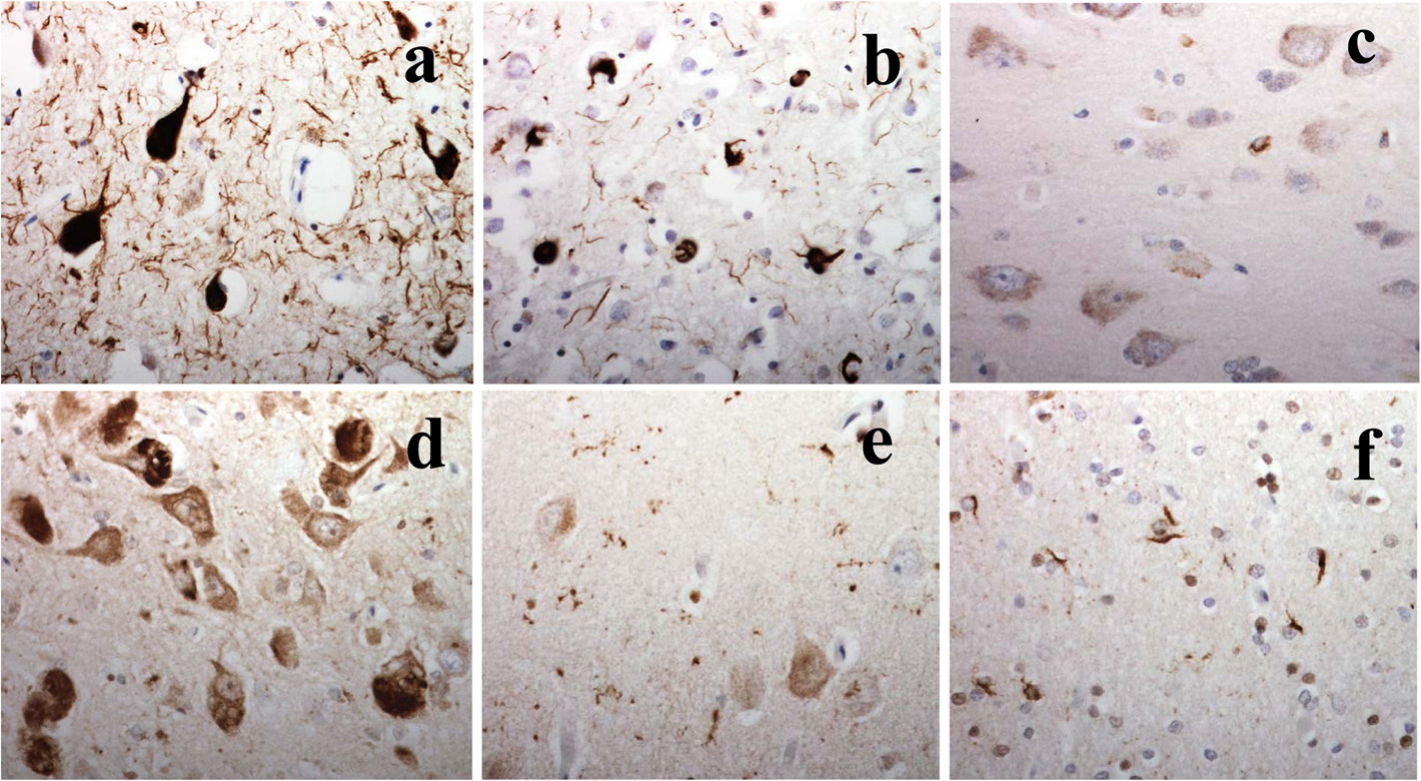
Neurofibrillary tangles and neuropil threads in entorhinal (**a**) and frontal (**b**) cortex in MND tau group 1 patients are immunoreactive for both 3-R (not shown) and 4-R tau (**a**, **b**) proteins. Amorphous tau deposits in neurones of frontal cortex in MND tau group 2 patients (patient #38) are 4-R (**c**), but not 3-R tau (not shown) immunoreactive. Neurofibrillary tangles and amorphous tau deposits in CA2 neurones in MND tau group 3 patients are both 4-R tau immunoreactive (**d**). Neurofibrillary tangles are also 3-R tau immunoreactive (not shown). Argyrophilic grains in CA1 region of hippocampus (**e**) and oligodendroglial tangles in underlying white matter (**f**) are both immunoreactive for 4-R tau (**e**, **f**) but not 3-R tau (not shown). Immunoperoxidase, x400 microscope magnification

In order to further characterise the molecular nature of the tau pathology present in each tau group, sections of frontal and/or temporal cortex from selected patients (ie tau group 1, patients #23, 31, 35, 36, 53, 56, 78 and 80; tau group 2 patients #37–40; tau group 3, patients #41 and 79) were subjected to immunostaining with 3-R (RD3) and 4-R (RD4) tau antibodies. These patients were selected because they showed the greatest levels of tau pathology within each of their respective groups, and were therefore considered to be most informative as regards the 3 patterns of tau pathology seen on AT8, pThr^175^ and pThr^217^ immunostaining. Sections of temporal and frontal cortex from tau group 1 cases showed neurofibrillary tangles, neuropil threads and neuritic plaques to be strongly immunoreactive for 4-R tau (Fig. 4a, b) and also, but less intensely so, for 3-R tau proteins (not shown). Sections of frontal cortex from tau group 2 cases showed neurones to be weakly immunoreactive for 3-R tau (not shown), but more strongly for 4-R tau protein (Fig. 4c). In tau group 3, there was strong 4-R tau immunostaining of neurofibrillary tangles and amorphous tau (pretangle) in cells of CA1 region, and amorphous tau staining in CA2 neurones, with tangles also being present in some CA2 cells (Fig. 4d). Tau grains were also strongly 4-R tau immunoreactive (Fig. 4e), as were oligodendroglial cells with coiled bodies in the adjoining white matter (Fig. 4f). The well-formed neurofibrillary tangles in CA2 region were also 3-R tau immunoreactive, but grains and glial cells were negative for 3-R tau (not shown).

**Fig. 5:**
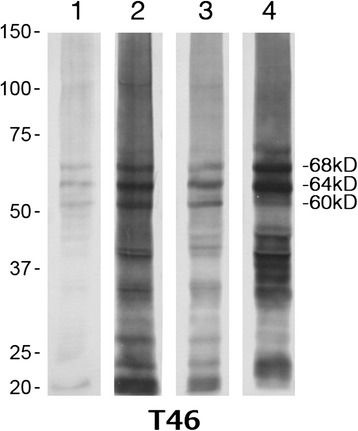
Immunoblot analysis of sarkosyl-insoluble tau extracted from temporal cortex, and detected by T46 (anti-tau C-terminus) antibody. MND tau group 1 (patients #30 and 35) (lanes 1 and 2, respectively) and group 2 (patient #40) (lane 3) show an Alzheimer’s disease-like triplet banding pattern comprising bands of hyperphosphorylated full-length tau at 60, 64 and 68 kDa, though various C-terminal fragments and smears are also detected. In contrast, the banding pattern in MND tau group 3 patients (patient #41) with Argyrophilic Grain Disease (lane 4) is characteristic of 4-R tauopathy with major bands at 64 and 68 kDa

Where available, frozen tissue samples of frontal and/or temporal cortex were taken from selected patients in each tau group (group 1, patients #23, 26, 30, 31, 34, 35, 55 and 80; group 2, patients #39 and 40; group 3, patient #41) and subjected to western blot analysis. Unfortunately, in most patients the amount of insoluble tau extractable from the tissue samples was too low to detect on blotting, even on 5-fold enrichment of applied sample. However, in patients #30 and 35 (tau group 1), 40 (tau group 2) and 41 (tau group 3) clear banding patterns were obtained from temporal, but not frontal, cortical samples which enabled molecular classification of the pathological tau proteins present (Fig. 5). Patients #30, 35 and 40 (lanes 1–3) showed an Alzheimer’s disease-like triplet banding pattern comprising bands of hyperphosphorylated full-length tau at 60, 64 and 68 kDa, though various C-terminal fragments and smears were also detected. In contrast, the banding pattern in patient #41 with AGD (lane 4) is characteristic of 4-repeat tauopathy with major bands at 64 and 68 kDa.

### Amyloid (4G8) immunostaining

On 4G8 immunostaining, 27 (66 %) patients with MND (patients #1–14, 18–26, 37–39 and 41, 15 (93 %) patients with FTD + MND (patients #42–56) and 17 (74 %) with FTD (patients # 58–68 and 71–76) showed no amyloid plaque pathology whatsoever in any brain region examined. Conversely, 14 (34 %) patients with MND (patients #15–17, 27–36 and patient #40) 1 (7 %) patient with FTD + MND (patient #57) and 6 (26 %) patients with FTD (patients #69, 70 and 77–80) displayed some degree of amyloid plaque pathology, however minimal. As with tau pathology, patients with MND were no more likely to display some amyloid plaque pathology than those with FTD + MND or FTD (χ2 = 4.25, *p* = 0.119). In 8 patients with MND (patients #16, 17, 27–30 and 40) and 2 with FTD (patients # 69 and 70) amyloid plaques were mostly or exclusively present as diffuse amyloid plaques, and only rare cored/neuritic plaques were generally present. Only in 6 patients with MND (patients #17, 31, 33–36) and 4 with FTD (patients #77–80) were neuritic plaques more widespread. Hence, the former 8 patients with MND and 2 with FTD were classed as A by CERAD criteria, the latter 6 with MND and 4 with FTD were classed as B. The single patient with FTD + MND showing amyloid pathology (patient #57) was classed as CERAD A.

Of the 14 patients with MND showing amyloid plaque pathology, two patients (patients #16 and 17) showed only mild neocortical involvement (usually temporal cortex, maximally) consistent with Thal phase 1. Two others (patients #28 and 40) showed allocortical involvement, consistent with Thal phase 2. Eight patients (patients #15, 27, 29, 30, 32–36) additionally showed amyloid deposition in the striatum (Thal phase 3) and one (patient#31) also showed brain stem and cerebellar involvement (Thal phase 5). The single patient with FTD + MND (patient #57) showed amyloid pathology at Thal phase 1. Of the 6 patients with FTD showing amyloid plaque pathology, four patients (patients #69, 70, 77 and 78) showed only mild to moderate neocortical involvement with mild allocortical involvement, consistent with Thal phase 2. The remaining 2 patients (patients #79 and 80) additionally showed mild amyloid deposition in the striatum (Thal phase 3), but neither also showed brain stem or cerebellar involvement.

Cerebral amyloid angiopathy (CAA) was generally absent in all 3 diagnostic groups, totally so in the FTD + MND group and affecting only a single patient in the FTD group). Occasional leptomeningeal arteries were affected in 4 MND patients (patients #15, 16, 29 and 36) though in 3 other MND patients (patients #17, 31 and 33) and the FTD patient with PNFA phenotype (patient #79) leptomeningeal CAA was more extensive, particularly in the occipital cortex, and in one of these (patient #33) this also involved capillaries within the primary visual cortex. In patient #79, cerebellar arteries were also affected.

### AT8 and 4G8 immunostaining

Fourteen of 41 patients with MND (34 %) (patients #1–14), 9/16 with FTD + MND (56 %) (patients #42–52) and 11/23 (48 %) patients with FTD (patients #58–68) showed neither amyloid nor tau pathology in any region of the brain examined. However, 11/41 (27 %) MND (patients #27–36 and patient #40) showed some degree of both tau and amyloid plaque pathology. Two of the patients (patients #28 and 40) showed Thal phase 2 amyloid plaque pathology, one of whom was Braak stage 0-I (patient #28), whereas the other (patient #40) was not classifiable by Braak staging. Eight other patients showed Thal phase 3 amyloid plaque pathology of which 1 patient was Braak stage 0-I, 1 was Braak stage I, 2 were Braak stage I-II, 2 were Braak stage II, 1 was Braak stage II-III and 1 was Braak stage V. Only 1 patient had a Thal phase greater than 3 (patient #31), but here Braak stage was only I-II. The single patient with FTD + MND showing both tau and amyloid plaque pathology (patient #57) was Thal phase 1 and Braak stage 1. Of the 4 patients with FTD with both tau and amyloid pathology (patients #77 and 78) were Thal phase 2, Braak stage 0-I, 1 (patient #80) was Thal phase 3, Braak stage I-II and the other (patient #79) was Thal phase 3, Braak stage IV-V.

Consequently, there were 13 patients with MND (patients #18–26, 37–39 and 41), 6 patients with FTD + MND (patients #51–56) and 6 patients with FTD (patients #71–76) that showed some degree of tau pathology but no amyloid plaque pathology at all. All of these were at Braak stage 0-I/I except patient #41 where no Braak classification was possible. Conversely, there were 3 patients with MND (patients #15–17) and 2 with FTD (patients #69 and 70) who showed amyloid plaque formation without tau pathology, 2 being at Thal phase 1 (patients #15 and 16) and 3 being at Thal phase 2 (patients #15, 69 and 70).

### Amyloid, tau, age and Apolipoprotein E (APOE) genotype

Where relevant age at onset and death data was available, patients with MND, MND + FTD or FTD, collectively, showing amyloid plaque formation were significantly older, both at onset (*p* = 0.003) and at death (*p* = 0.001), than those not showing amyloid plaque formation. However, duration of illness did not differ between each group (*p* = 0.343) (Table 2). By contrast, there were no significant differences between patients with MND, MND + FTD or FTD, collectively, showing any type of tau pathology and those without tau pathology at all, for age at onset (*p* = 0.493), age at death (*p* = 0.726) or duration of illness (*p* = 0.364) (Table 2).

*APOE* genotype was only available for 65/80 patients. Overall, the *APOE* ε4 allele frequency was 12.5 % (16/128 alleles), and was not significantly different in MND (10.7 %, 6/56 alleles), FTD + MND (13.3 %, 4/30 alleles) or FTD (13.6 %, 6/44 alleles (χ2 = 0.232, *p* = 0.890), although the *APOE* ε4 allele frequency in those patients with MND, FTD + MND and FTD, collectively, showing amyloid plaque formation (20 %, 8/40 alleles) tended to be significantly greater (χ2 = 3.17, *p* = 0.075) than in those without amyloid (8.9 %, 8/90 alleles).

Nonetheless there was a significant effect of age. Irrespective of *APOE* genotype, 14 of the 15 patients showing amyloid plaques were all over 65 years of age at death. Conversely, only 23 of the 53 patients without amyloid were over 65 years of age at death, and of these only 3 bore *APOE* ε4 allele. The other 5 *APOE* ε4 allele bearers not showing amyloid plaque formation were all under 65 years of age at death. Patients with MND, MND + FTD and FTD, collectively, were therefore significantly more likely to show amyloid in the brains if they died after the age of 65 years (χ2 = 11.7, *p* < 0.001). Indeed, all except 1 of the 10 patients with MND, MND + FTD and FTD, collectively, who showed both amyloid and tau in their brains were over 65 years of age at death, and of these 9 patients, 6 were bearers of *APOE* ε4 allele. Hence, patients with MND, MND + FTD and FTD most likely to show amyloid in their brains were those who died after the age of 65 years and bore *APOE* ε4 allele.

## Discussion

Although the presence of some degree of tau pathological changes in patients with ALS/MND [16, 29] or FTLD [29] has been anecdotally reported, there has been only little work where this has been systematically studied. Using novel antibodies to tau phosphorylated at Ser208/210, Thr175 and Thr217, Yang and Strong [36] investigated 5 MND patients with cognitive impairment (ALSci) and 5 others with no cognitive impairment (ALS) (as defined by Strong et al [31]). In the ALS patients, they observed a limited number of intraneuronal tau inclusions (neurofibrillary tangles) and neuropil threads in temporal lobe structures, mostly in entorhinal cortex, and amygdala, less so in hippocampus and frontal and cingulate cortex in 1–3/5 cases, which were broadly similarly immunoreactive with all 3 antibodies. A similar type of tau pathology was seen in the entorhinal cortex, amygdala and hippocampus in the 5 ALSci cases, but in these the frontal cortex and cingulate gyrus were more often involved (usually in 3–5/5 cases). Again, the level of immunostaining with all 3 antibodies was roughly similar. Although, Yang and Strong [36] did not perform Braak staging for neuronal tau, from their descriptions it can be inferred that cases of ALS were at Braak stages 0/I, whereas those with ALSci may have been at Braak stages III-IV.

In the present study we have shown there to be ‘significant’ neuronal tau pathology in 59 % patients with MND, 44 % patients with FTD + MND and 44 % patients with FTD, whereas some degree of amyloid pathology was present in only 34 % patients with MND, 7 % patients with FTD + MND and 26 % patients with FTD. In this study, we have also employed the same antibodies to tau phosphorylated at Thr175 and Thr217, along with commercial AT8 antibody, and have supplemented these observations with 3-R and 4-R tau immunostaining and western blotting, on selected patients. Analysis of the patterns of tau and amyloid plaque pathologies suggested several ‘profiles’ to be present.

Firstly, in those 9 patients with MND (patients #18–26), 6 with MND + FTD (patients #51–56) and 6 with FTD (patients #71–76), where minimal temporal lobe tau (Braak stage 0-I/I) but no amyloid plaque pathologies were present, the tau changes might be simply considered to be ‘age-related’ and unlikely to be associated with (early stage) Alzheimer’s disease, given the lack of amyloid pathology [23]. Nonetheless, the concept of Primary Age-Related Tauopathy (PART) has been promoted to describe cases where tau pathology, especially medial temporal lobe tau, occurs in the complete absence of amyloid plaque deposition (Thal phase zero), or at least minimal amounts [10, 15]. Such a designate would encompass pathologies formerly described as ’tangle only dementia’, or ‘tangle predominant senile dementia’ where extensive tau pathology, but usually not beyond Braak stage III-IV, is seen (sometimes) in the presence of an identifiable dementia or cognitive impairment [5, 15, 35]. Unfortunately, because of the low level of tau pathology present, and despite a 5-fold enrichment of sample, it was not possible to demonstrate any tau banding patterns on western blot in either frontal or temporal cortex in any of patients which might illuminate the molecular nature of this staining. Nonetheless, the neurofibrillary changes present were detected by both 3-R and 4-R tau immunostaining, as is typical for Alzheimer’s disease, and as has been reported in PART by others [15]. Consequently, these 21 patients with limited temporal lobe tau pathology, but no amyloid, might alternatively be considered to fall under the ‘umbrella’ of PART.

Secondly, in those 10 patients with MND (patients # 27–36), 1 with FTD + MND (patient #57) and 3 with FTD (patients #77–79), where both tau AND amyloid pathology was present, the pattern and distribution of tau pathology within the temporal lobe (and other regions when present) was of the type associated with Alzheimer’s disease, ie neurofibrillary tangles, neuropil threads and occasionally neuritic plaques. However, in most instances the extent of neurofibrillary pathology clearly fell well short of that associated with fully developed Alzheimer’s disease, and none of the patients met pathological diagnostic criteria for (a high probability of) Alzheimer’s disease [23]. For the most part, this can be interpreted as ‘incidental’ and probably-age related, being of that type commonly seen in many older healthy, individuals and considered unlikely to generate significant clinical dysfunction [8]. Nonetheless, three patients did meet pathological criteria for an intermediate likelihood of Alzheimer’s disease [23]. One of these patients (patient#36) showed mild cognitive impairment, another (patient #35) also had isocortical DLB and was clinically demented, and the third (patient #79) had FTD (with PNFA). Where tau pathology was sufficiently extensive to make western blotting possible (in patients #30 and 35) this produced a banding pattern consistent with Alzheimer’s disease, and neurofibrillary changes were detected by 3-R and 4-R tau immunostaining, again consistent with (an evolving) Alzheimer’s disease pathology.

Thirdly, in 4 patients (patients #37–40) an unusual pattern of tau pathology (fine or coarse granules) was seen, which was only demonstrated by pThr^175^ immunostaining, and not at all with pThr^217^ or AT8 antibodies. Such changes were most prominent in frontal cortex, being uncommon in, or absent from, temporal cortex. None of the 4 patients were considered to have shown overt clinical evidence of cognitive impairment, although this had not been formally assessed in any of the 4 patients. Again, despite 5-fold enrichment of sample, it was not possible in patients #37–39 to demonstrate on western blot from the frontal cortex any tau banding pattern relevant to the pThr^175^ tau pathology seen histologically in the frontal cortex of these patients. However, in patient #40 a pattern resembling that of Alzheimer’s disease was seen in the temporal cortex sample consistent with the presence of limited tau neurofibrillary tangle formation and mild amyloid deposition on histological inspection. Immunostaining for both 3-R and 4-R tau showed occasional nerve cells in frontal cortex to be immunoreactive for both, again consistent with the presence of mild Alzheimer-type pathology within temporal lobe only. Consequently, the nosology, and significance, of the pThr^175^ frontal cortical tau pathology, of these cases (including case #40) presently remains uncertain.

Yang and Strong reported the presence of tau-immunoreactive astrocytes, especially within the frontal cortex, amygdala and entorhinal cortex, that were generally much more common in ALSci than ALS, and were more strongly detected using pThr^217^ antibody than pThr^175^ or pSer^208/210^ antibodies [36]. From descriptions presented, it is difficult to ascertain precisely just how common this glial cell pathology might have been, but from inspection of the tabulated data, it would appear to be sparse in any region of brain in ALS in most patients, being relatively frequent only in isolated individuals (in 1/5 studied). In ALSci tau positive glial cells were seen in a greater proportion of patients (at least in frontal cortex), but were not seemingly present in any greater numbers than in ALS alone. In the present study, tau positive astrocytes cells were not, or only very rarely, seen irrespective of diagnosis, these being equally detected by AT8, pThr^175^ and pThr^217^ antibodies. The reasons for this discrepancy are not clear, but may relate to case selection or tissue processing. In the present study, cases of MND, FTD + MND and FTD were unselected, representing consecutive cases entering Manchester Brain Bank from 1986 onwards. The MND patients, with the exception of two, were not thought to exhibit cognitive change, although in the absence of formal neuropsychological assessments, the presence of subtle changes cannot be excluded. Patients with FTD + MND and FTD had undergone extensive neuropsychological assessment and their pattern of behavioural, personality and cognitive change was well documented [30, 34]. The degree of clinical and pathological overlap between the ALSci cases reported by Yang and Strong [36] and those in the current series is open to debate.

Hence, in the present study, we were able to substantiate Yang and Strong’s findings of neuronal/neuritic tau pathology in over half of patients with MND, this also being similarly present in around 40 % of FTD + MND and FTD. The tau pathology was of a type similar to that seen in Alzheimer’s disease, albeit to a much more limited extent, usually confined to temporal lobe structures, sometimes restricted to entorhinal cortex. The clear inference from present observations is that when cognitive impairment does occur in MND, this is most likely to be associated with Alzheimer’s disease pathology, particularly involving medial temporal lobe structures. Exacerbation of this extent of pathology in ALS/MND might explain the cognitive deficits seen in patients with ALSci reported by Yang and Strong [36].

## Conclusions

Nonetheless, if it were to be accepted that FTD, FTD + MND and MND exist on a continuum, and that the clinical combination of FTD + MND can be driven in either direction from FTD or MND through a common pathogenetic pathway, then it might have been anticipated that a neuronal and/or glial cell tauopathy, similar to that reported by Yang and Strong [36], would have been (more widely) present in our FTD + MND, even more so in FTD, than MND alone. In fact, the extent of neuronal tau in FTD + MND, in line with that seen in FTD, was not greater, but in fact less, than that seen in MND alone, and a glial cell tau pathology specifically detected by pThr^217^ immunostaining in MND [36] was not seen in either FTD + MND or FTD alone. Consequently, present data suggests that the route to cognitive impairment in at least some cases of MND (ie ALSci) may be dissimilar to that seen in FTD (and leading to FTD + MND), which is most likely associated with TDP-43 proteinopathy in both conditions, thereby challenging the notion that all cognitive changes in FTD, FTD + MND and MND exist on a common pathological continuum. The presence of tau pathology in MND patients should not be considered a marker of a person’s likelihood of developing FTD.

## Additional files

Additional file 1: Figure S1. α-synuclein (a-c) pathology in cingulate gyrus (a), CA2 region of hippocampus (b) and substantia nigra (c) and TDP-43 pathology in anterior horn cells of the spinal cord (d-f), with fine, particulate accumulations of TDP-43 (d,e) or skein-like structures (e,f) being present in affected cells in which the nucleus has been ‘cleared’ of its normal immunoreactivity. Immunoperoxidase, x400 microscope magnification. (DOCX 6504 kb) Additional file 2: File S1. Clinical histories and neuropathological findings in patients #35 and 41. (DOCX 21 kb)
