# Supplementary figures and images for: Pathological tau deposition in Motor Neurone Disease and frontotemporal lobar degeneration associated with TDP-43 proteinopathy
Source: Acta Neuropathol Commun. 2016 Mar 31;4:33. doi: 10.1186/s40478-016-0301-z (PMC4818389; doi:10.1186/s40478-016-0301-z)

**f**

**e**

**d**

**c**

**b**

**a**


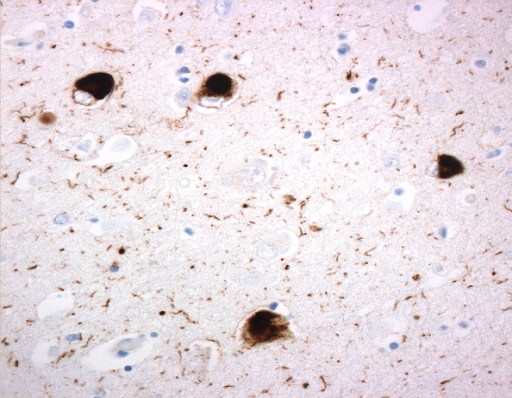

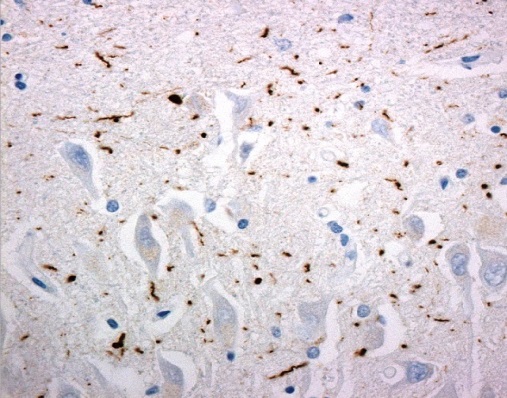

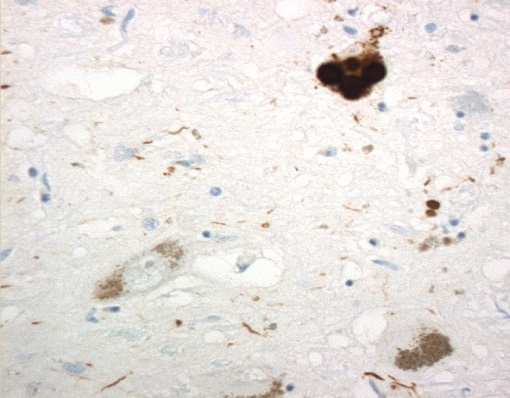

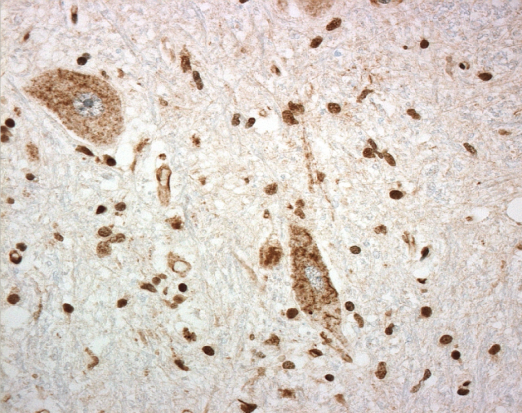

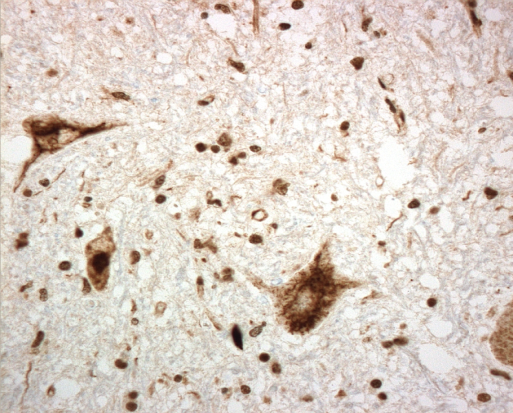

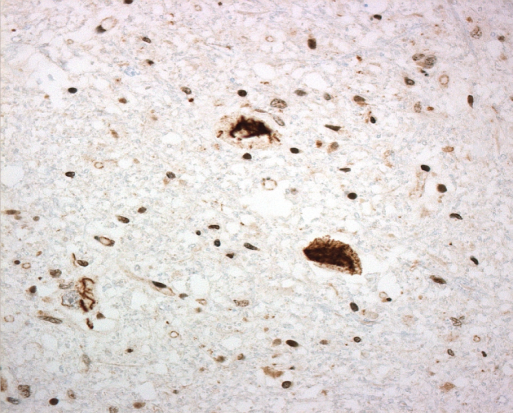

Supplement: Additional file 1: Figure S1. — α-synuclein (a-c) pathology in cingulate gyrus (a), CA2 region of hippocampus (b) and substantia nigra (c) and TDP-43 pathology in anterior horn cells of the spinal cord (d-f), with fine, particulate accumulations of TDP-43 (d,e) or skein-like structures (e,f) being present in affected cells in which the nucleus has been ‘cleared’ of its normal immunoreactivity. Immunoperoxidase, x400 microscope magnification. (DOCX 6504 kb) [file 40478_2016_301_MOESM1_ESM.docx]
