# Supplementary material for: Pathological tau deposition in Motor Neurone Disease and frontotemporal lobar degeneration associated with TDP-43 proteinopathy
Source: Acta Neuropathol Commun. 2016 Mar 31;4:33. doi: 10.1186/s40478-016-0301-z (PMC4818389; doi:10.1186/s40478-016-0301-z)
Supplement: Additional file 2: — File S1. Clinical histories and neuropathological findings in patients #35 and 41. (DOCX 21 kb) [file 40478_2016_301_MOESM2_ESM.docx]

**Patient Histories and Neuropathological Findings**

***Patient #35***

This lady was referred to Cerebral Function Unit, Salford Royal Hospital, in 2012, aged 69 years, with 12-18 month progressive history of reduced speech output, and impaired understanding with loss of emotion, becoming more childlike. She was in the habit of pacing the room and neglected self-care. She had difficulty swallowing, with excessive salivation. She had a previous medical history of Crohn’s disease and diabetes. Her father had suffered from dementia in old age, but died at 95 years.

On examination, she was distractible and difficult to engage. She was anarthric, tongue movements were slowed and jaw jerk was brisk. She showed impairment of executive functions being impulsive, distractible, inattentive, impersistent, with poor generation and perseveration. MR showed generalised brain atrophy. Frontal lobe syndrome and pseudobulbar palsy consistent with FTD/MND was diagnosed. She died aged 69 years, 2 years after onset of symptoms.

At post mortem, the brain weighed 1071g. The major cerebral arteries appeared normal. The cerebral hemisphere showed moderate frontal, but mild temporal, lobe atrophy. The motor cortex also appeared mildly atrophic. The cerebellum and brain stem appeared externally normal. On coronal section, the lateral ventricle was slightly enlarged with rounding at the angle, though the temporal horn was of normal size. There was moderate atrophy of premotor (frontal) and motor cortex but all other cortical regions, including temporal lobe, appeared normal. The distinction between grey and white matter was well maintained throughout, and the white matter appeared uniformly well myelinated. The corpus callosum was of normal thickness. The hippocampus and amygdala both appeared normal, as did all basal ganglia regions. The substantia nigra and locus caeruleus were both conspicuously underpigmented. The rest of the brain stem and cerebellum appeared normal. No haemorrhages or infarctions were seen, and no vascular abnormalities were present.

The entorhinal cortex, fusiform cortex, inferior temporal, superior and middle temporal and superior frontal cortex, however, showed moderate densities of neurofibrillary tangles, neuropil threads and occasional neuritic plaques, whereas the striate and peristriate occipital cortex was spared. The hippocampus showed only limited amounts of neurofibrillary tangles and neuropil threads, chiefly within CA1 and subicular regions. No significant glial tau-related pathology was identified in white or grey matter. A high density of beta amyloid plaques were seen in all neocortical regions, with fewer plaques in the hippocampus. Alpha-synuclein immunostaining demonstrated moderate to high densities of cortical Lewy bodies in both frontal and temporal neocortex. A high density was also identified in the collateral fissure in the medial temporal lobe with immunopositive neurites present in CA2/3 in the hippocampus. TDP-43 immunostaining demonstrated occasional cytoplasmic inclusions in the granular neurons of the dentate gyrus in the hippocampus. The cerebral cortex, including frontal and temporal cortex, as well as motor cortex, did not show any significant TDP-43 related pathology in the form of neuronal inclusions or neurites.

The substantia nigra showed readily identifiable alpha-synuclein immunopositive Lewy bodies, and a low density of neurites. A few tau-immunopositive globose neurofibrillary tangles, some neuropil threads, were also seen. The locus caeruleus also showed low to moderate numbers of neurofibrillary tangles and neuropil threads. The motor nuclei within the pons and medulla (including the trigeminal and hypoglossal nuclei) contained skein-like ubiquitin and TDP-43 immunopositive inclusions consistent with MND-type inclusions. At the upper cervical/cervicomedullary junction, anterior horn cells also showed similar skein-like MND inclusions. The cerebellum appeared normal. The deep grey matter (basal ganglia and thalami) structures showed mild hyaline arteriolosclerosis with some limited perivascular rarefaction. No significant neurodegenerative changes were identified in these regions. P62 immunostaining did not reveal any TDP-43 negative inclusions within dentate gyrus or cerebellar granule cells, or within CA4 neurones of the hippocampus. The Apolipoprotein E genotype was ε2ε3. No expansion in C9orf72 gene was detected.

# *Patient #41*

The patient was referred to the Manchester MND Care Centre, aged 52 years. He reported a 2-year history of progressive speech and swallowing disturbance alongside muscle twitching. There was no suggestion of cognitive impairment or family history of neurodegenerative disease. On examination, he had a pseudobulbar palsy and an associated pseudobulbar affect, widespread limb and trunk fasciculation and increased tone in the left lower limb.

Routine investigations and brain imaging were normal. However, neurophysiology (EMG/NCS) was consistent with motor neurone disease.

He was started on riluzole, his pseudobulbar affect improved on citalopram and a gastrostomy tube was inserted. Within 2 months of diagnosis he was reporting dyspnoea and investigations confirmed nocturnal hypoventilation. He was referred for non-invasive ventilation that was well tolerated despite his pseudobulbar disease. His motor disease progressed and he died 17 months after diagnosis without developing any obvious cognitive difficulties.

At post mortem, the brain weighed 1240g. The major cerebral arteries were free from atheroma. The cerebral hemispheres, cerebellum and brainstem were without external abnormality. On coronal section, the lateral ventricle was mildly dilated but the temporal horn was of normal size. All regions of the cerebral cortex appeared normal with a clear demarcation between grey and white matter, which was well myelinated. The hippocampus and amygdala both appeared normal. The corpus callosum was normal at all levels. The corpus striatum and thalamus showed no abnormality. Substantia nigra and locus caeruleus were both reasonably pigmented. The rest of brainstem and cerebellum appeared normal on section. No cerebrovascular changes were seen, and no overt haemorrhages or infarctions were present.

All neocortical areas examined (including frontal, temporal, cingulate, inferior parietal and visual association cortex) showed complete absence of amyloid plaques and amyloid angiopathy. Tau immunostaining revealed a moderate number, or many, neurofibrillary tangles, neuropil threads and dystrophic neurites within the hippocampus (CA1 and subiculum mostly), amygdala, entorhinal and temporal neocortex. Frontal and anterior parietal areas of neocortex show only sparse neurofibrillary changes, and occipital and posterior parietal cortex, none. Additionally there were moderate numbers of tau-immunoreactive coiled bodies within glial cells of the temporal lobe white matter, but these were sparse within frontal and cingulate cortex, and absent from inferior parietal and occipital cortex. Hippocampal sclerosis of CA1 was not present. There was moderate neuronal loss from the substantia nigra with occasional cells showing pigment incontinence. No obvious Lewy bodies or tau tangles were seen. TDP-43 immunoreactive inclusions (mostly as skeins) were present in surviving anterior horn cells in the upper cervical cord, and rarely in motor neurones of the trigeminal and hypoglossus nuclei. TDP-43 inclusions were widely present (as granular structures) within the dentate gyrus granule cells, but less commonly as granular accumulations within pyramidal cells of layer 2 of frontal and temporal cortex. No fully developed neuronal cytoplasmic inclusions, dystrophic neurites or intranuclear inclusions were seen. P62 immunostaining did not reveal any TDP-43 negative inclusions within dentate gyrus or cerebellar granule cells, or within CA4 neurones of hippocampus. No vascular abnormalities were seen. The Apolipoprotein E genotype was ε3ε3. No mutation in MAPT or expansion in C9orf72 was detected.
